# Palatal rugoscopy: Establishing identity

**DOI:** 10.4103/0974-2948.71054

**Published:** 2010

**Authors:** Aparna Paliwal, Sangeeta Wanjari, Rajkumar Parwani

**Affiliations:** *Department of Oral and Maxillofacial Pathology, Modern Dental College and Research Centre, Indore (M.P.), India*

**Keywords:** Identification, palatal rugae, rugoscopy

## Abstract

Palatal rugae are irregular, asymmetric ridges of the mucous membrane extending laterally from the incisive papilla and the anterior part of the palatal raphe. The uniqueness and the overall stability of palatal rugae suggest their use for forensic identification.

**Objective:**

The purpose of this study was to compare the palatal rugae patterns in 2 different populations in India (Madhya Pradesh and Kerala), and furthermore, to assess the predominant pattern if any in the selected groups.

**Materials and Methods:**

60 maxillary study models (30 from each group) were examined in the age group ranging from 17 to 23 years. Palatal rugae pattern were examined in both the sexes on right and left sides of the palate for the total number (quantitative), length, shape, and predominant direction (qualitative).

**Results:**

After analyzing the rugae patterns in both the groups and between the 2 sides of the palate, the wavy pattern was found to be predominant followed by curved, straight, unification, circular, and nonspecific in decreasing order in the overall population.

**Conclusion:**

Straight rugae pattern on the right side of the palate in the male subjects was found to be significantly predominant in the MP population, whereas wavy shape was predominant in Keralites; however, rugae patterns on the right side of the palate in female subjects exhibited no significant difference.

## Introduction

Establishing a person’s identity can be a very difficult process in forensic identification. Dental, fingerprints, and DNA comparisons are the most common techniques used in this context allowing fast and secure identification. However, since they cannot be always used, sometimes simple techniques can be used successfully in human identification, such as ‘Palatal rugoscopy,‘ which is the study of palatal rugae.[1] Palatal rugae have been equated with fingerprints and are unique to an individual.[2–5] It can be of special interest in edentulous cases and also in certain conditions where there are no fingers to be studied, such as burned bodies or bodies that underwent severe decomposition.[1] Rugae pattern may be specific to racial groups facilitating population identification. Thus the uniqueness, postmortem resistance, overall stability, and additionally low utilization cost makes palatal rugae an ideal forensic identification parameter.[2] The present study aims to determine the number and pattern of palatal rugae in 2 different populations in India and also to assess the predominant pattern if any in the selected groups.

## Materials and Methods

**Figure 1:**
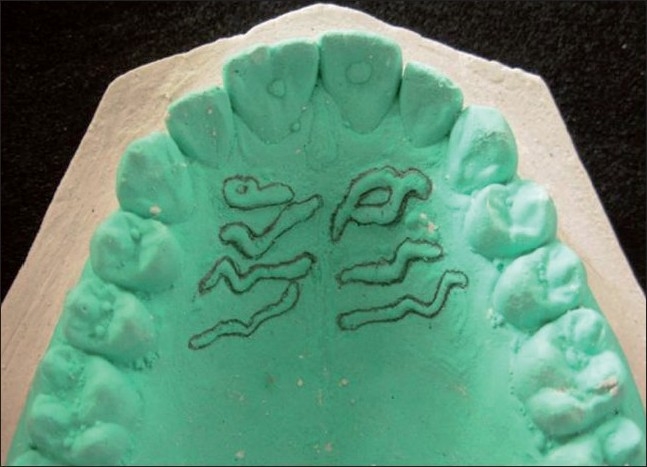
Cast showing tracing of palatal rugae pattern

The study consisted of 60 subjects, 30 each from the 2 groups of geographically different regions of India, namely, Madhya Pradesh (MP) and Kerala. The sample size was equally distributed among both the sexes in the age range of 17–23 years. After obtaining informed consent, alginate impression of maxillary arch was made and the study models were prepared in dental stone for interpretation. The rugae were delineated using a sharp graphite pencil and recorded [Figure 1] according to the classification given by Thomas and Kotze (1983).[6] After determining the length of all the rugae, we considered 2 categories:

1. Primary rugae: more than 5 mm
2. Secondary rugae: 3–5 mm.

**Figure 2:**
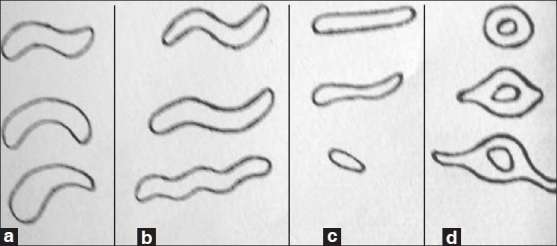
Classification of rugae based on shape (a) curved (b) wavy (c) straight (d) circular

The shapes of individual rugae were classified into 4 major types: curved, wavy, straight, and circular [Figure 2]. Straight types ran directly from their origin to insertion. The curved type had a simple crescent shape with a gentle curve. Wavy rugae were serpentine in shape and rugae that showed definite continuous ring formation were classified as circular. Additionally, nonspecific rugae pattern was observed, which did not fall in any of the mentioned classes.

**Figure 3:**
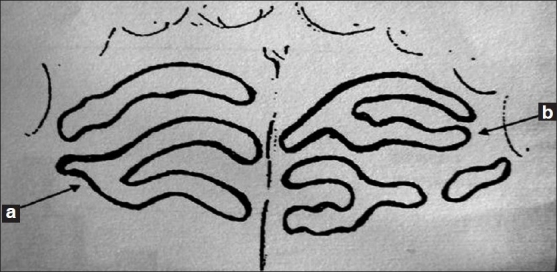
Classification of rugae based on unification (a) converging and (b) diverging rugae

Unification occurs when 2 rugae are joined at their origin or termination. Rugae were considered diverging if 2 rugae had the same origin but immediatey branched, whereas rugae with different origins, which joined on their lateral portions were considered converging [Figure 3].[3,7] In the present study, only unification was reported without further categorizing as converging or diverging.

The association between rugae shape and ethnicity was tested using Chi-square analysis and comparison between the 2 groups for different parameters was done using Student *t* test.

## Results

**Figure 4:**
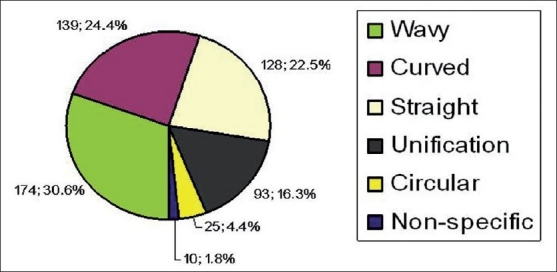
Distribution of total rugae shapes in total subjects of Madhya Pradesh and Kerala

The total number of rugae in the 2 populations and between the 2 sides of the palate showed no statistically significant difference [Table 1]. On observing the length, primary rugae were predominant as compared with secondary rugae. Furthermore, Keralite females showed slightly more number of secondary rugae as compared with MP females [Table 2]. The distribution of rugae shape in the overall population was seen as wavy pattern to be 30.6% followed by curved 24.4% and straight 22.5%. Unification constituted 15.4%. Circular forms were also seen (4.4%), whereas nonspecific constituted only 1.8% [Figure 4].

**Table 1:** Distribution of rugae numbers in Madhya Pradesh and Kerala subjects

Group	Males				Females				Total			
	MP Mean±SD	Kerala Mean±SD	*t* Value	*P*>0.05	MP Mean±SD	Kerala Mean±SD	*t* Value	*P*>0.05	MP Mean±SD	Kerala Mean±SD	*t* Value	*P*>0.05
Total number	9.50±1.71	9.18 ± 1.81	0.520	0.606 NS	9.50±1.70	9.69 ± 1.89	0.278	0.783 NS	9.50±1.68	9.40±1.83	0.212	0.832 NS
Right	4.88±1.15	4.88±1.11	0.019	0.984 NS	4.71±1.33	5.00±1.08	0.610	0.547 NS	4.80±1.21	4.93 ± 1.08	0.449	0.655 NS
Left	4.63 ± 1.09	4.29 ± 1.10	0.860	0.396 NS	4.79±0.97	4.69 ± 1.11	0.232	0.819 NS	4.70±1.02	4.47 ± 1.11	0.848	0.399 NS

NS=Nonsignificant

**Table 2:** Distribution of rugae length (primary and secondary rugae) in Madhya Pradesh and Kerala subjects

Group	Males				Females				Total			
	MP Mean±SD	Kerala Mean±SD	*t* Value	*P*>0.05	MP Mean±SD	Kerala Mean±SD	*t* Value	*P*>0.05	MP Mean±SD	Kerala Mean±SD	*t* Value	*P*>0.05
Primary rugae	8.38 ± 1.50	8.06 ± 1.25	0.659	0.514 NS	8.21±1.42	8.38 ± 1.45	0.308	0.760 NS	8.30±1.44	8.20±1.32	0.279	0.781 NS
Secondary rugae	1.13±0.79	1.12±0.81	0.019	0.850 NS	0.86±0.56	1.38±0.96	1.671	0.107 NS	1.00±0.61	1.23±1.04	0.924	0.359 NS

NS=Nonsignificant

As shown in Table 3, in the total subjects (male ± female) wavy pattern was predominant followed by straight and curved in the MP group, whereas wavy was followed by curved and then straight in the Kerala group. Overall in males, wavy and straight patterns were predominant in the MP group, whereas wavy was followed by curved pattern in the Kerala group. However, in females wavy pattern was predominant, whereas straight and curved pattern were at par with each other in both the groups. As shown in Table 4, straight-shaped rugae pattern for right side of the palate in the male subjects was significantly more in the MP group (*t*=2.1; *P*<0.05), whereas wavy shape was predominant in Keralite males (*t*=2.09; *P*<0.05). Similarly, statistically significant differences were seen in the total subjects of the 2 groups with regard to the patterns on the right side as follows: straight pattern was found to be predominant in the MP group (*t*=2.08; *P*<0.05), whereas wavy pattern was found to be more prevalent in Keralites (*t*=2.00; *P*<0.05). In females from MP state, straight and wavy shapes were at par with each other, whereas wavy and curved shapes were almost the same among Keralite females, as shown in Table 5. On evaluating the left side, wavy pattern was more predominant in males and females as well as in total subjects in both the populations [Table 6].

**Table 3:** Distribution of total number of different Rugae shapes in total subjects of Madhya Pradesh and Kerala

Rugae shapes	Males			Females			Total		
	MP	Kerala	Chi-square value (*P*)	MP	Kerala	Chi-square value (*P*)	MP	Kerala	Chi-square value (*P*)
Straight	38 (12.3)	25 (8.1)	2.29 (0.130)	36 (13.8)	29 (11.1)	0.55 (0.458)	74 (13.0)	54 (9.5)	2.82 (0.458)
Wavy	43 (14.0)	54 (17.5)	1.03 (0.310)	41 (15.7)	36 (13.8)	0.21 (0.646)	84 (14.8)	90 (15.8)	0.14 (0.646)
Curved	35 (11.4)	39 (12.7)	0.12 (0.729)	36 (13.8)	29 (11.1)	0.55 (0.458)	71 (12.5)	68 (12.0)	0.03 (0.458)
Unification	25 (8.1)	27 (8.8)	0.02(0.887)	18 (6.9)	23 (8.8)	0.39 (0.532)	43 (7.6)	50 (8.8)	0.39 (0.532)
Circular	9 (2.9)	8 (2.6)	0.00 (1.000)	2 (0.8)	6 (2.3)	1.13 (0.287)	11 (1.9)	14 (2.5)	0.16 (0.287)	
Nonspecific	2 (0.6)	3 (1.0)	0.00 (1.000)	2 (0.8)	3 (1.1)	0.00 (1.000)	4 (0.7)	6 (1.1)	0.10 (1.000)	
Chi-square value for									
Rugae shapes		4.43 NS			4.34 NS			4.64 NS		
Regions		0.06 NS			0.31 NS			0.05 NS		

Parentheses=%; P=Probability; NS=Nonsignificant

**Table 4:** Significant parameters for rugae shapes in Madhya Pradesh and Kerala samples

Group	Males			Females			Total		
	MP Mean±SD	Kerala Mean±SD	*t* Value	MP Mean±SD	Kerala Mean±SD	*t* Value	MP Mean±SD	Kerala Mean±SD	*t* Value	
Straight right	1.38±0.72	0.76±0.56	2.14*S	1.36±1.01	1.08 ± 0.86	0.773 NS	1.37 ± 0.85	0.90±0.68	2.08*S	
Wavy right	1.13±0.82	1.88±0.65	2.09*S	1.36±1.01	1.38±0.87	0.075 NS	1.23±1.01	1.67±0.99	2.00*S	

* S=Significant; NS=Nonsignificant

**Table 5:** Distribution of total number of different rugae shapes on the right side of Madhya Pradesh and Kerala subjects

Rugae shapes	Males			Females			Total		
	MP	Kerala	Chi-square value (*P*)	MP	Kerala	Chi-square value (*P*)	MP	Kerala	Chi-square value (*P*)
Straight	22 (13.7)	13 (8.1)	1.83 (0.176)	19 (14.4)	14 (10.6)	0.48 (0.488)	41 (14.0)	27 (9.2)	2.49 (0.114)
Wavy	18 (11.2)	32 (19.9)	3.38 (0.065)	19 (14.4)	18 (13.6)	0.00 (1.000)	37 (12.6)	50 (17.1)	1.66 (0.197)
Curved	18 (11.2)	17 (10.6)	0.00 (1.000)	17 (12.9)	19 (12.9)	0.03 (0.862)	35 (11.9)	36 (12.3)	0.00 (1.000)
Unification	17 (10.6)	15 (9.3)	0.03 (0.479)	11 (8.3)	11 (8.3)	0.05 (0.823)	28 (9.6)	26 (8.9)	0.02 (0.887)
Circular	3 (1.9)	4 (2.5)	0.00 (1.000)	1 (0.8)	2 (1.5)	0.00 (1.000)	4 (1.4)	6 (2.0)	0.10 (0.751)
Nonspecific	0 (0.0)	2 (1.2)	0.50 (0.479)	0 (0.0)	1 (0.8)	0.00 (1.000)	0 (0.0)	3 (1.0)	1.33 (0.248)
Chi-square value for									
Rugae shapes		8.38 NS			2.20 NS			8.28 NS	
Regions		0.10 NS			0.04 NS			0.03 NS	

Parentheses=% P=Probability; NS=Nonsignificant

**Table 6:** Distribution of total number of different rugae shapes on the left side of Madhya Pradesh and Kerala subjects

Rugae shapes	Males			Females			Total		
	MP	Kerala	Chi-square value (*P*)	MP	Kerala	Chi-square value (*P*)	MP	Kerala	Chi-square value (*P*)
Straight	16 (10.9)	12 (8.2)	0.32 (0.571)	17 (13.2)	15 (11.6)	0.03 (0.862)	33 (12.0)	27 (9.8)	0.42 (0.516)
Wavy	25 (17.0)	22 (15.0)	0.09 (0.764)	22 (17.1)	18 (14.0)	0.23 (0.631)	47 (17.0)	40 (14.5)	0.41 (0.522)
Curved	17 (11.6)	22 (15.0)	0.41 (0.521)	19 (14.7)	10 (7.8)	2.21 (0.137)	36 (13.0)	32 (11.6)	0.13 (0.718)
Unification	8 (5.4)	12 (8.2)	0.45 (0.502)	7 (5.4)	12 (9.3)	0.84 (0.359)	15 (5.4)	24 (8.7)	1.64 (0.200)
Circular	6 (4.1)	4 (2.7)	0.10 (0.751)	1 (0.8)	4 (3.1)	0.80 (0.371)	7 (2.5)	8 (2.9)	0.00 (1.000)
Nonspecific	2 (1.4)	1 (0.7)	0.00 (1.000)	2 (1.6)	2 (1.6)	0.25 (0.617)	4 (1.4)	3 (1.1)	0.00 (1.000)
Chi-square value for									
Rugae shapes		2.93 NS			6.07 NS			3.46 NS	
Regions		0.01 NS			0.39 NS			0.43 NS	

Parentheses=% P=Probability; NS=Nonsignificant

## Discussion

Palatal rugoscopy was first proposed in 1932, by a Spanish investigator Troban Hermaso. Since then various classifications had been given. Most studies are based on systems devised by Lysell and Thomas and Kotze, although they may differ in detail.

In the literature, the consensus of opinion is that the rugae remain fairly stable in number and morphology except when there is trauma, such as loss of tooth, persistent pressure, extreme finger sucking, orthodontic tooth movement, which may modify the alignment.[3,8] Thomas and Kotze (1983) studied the rugae patterns of 6 South African populations to analyze the interracial difference. They found that rugae were unique to each ethnic group and that it can be used successfully as a medium for genetic research.[6]

Hauser *et al*. (1989) compared the rugae pattern of Swazi and Greek populations and found definite differences in the rugae pattern between the 2 populations. It was seen that the degree of development of rugae was dependent on the growth of the palate.[8] According to English *et al*. (1988), palatal rugae pattern is sufficiently characteristic to discriminate between individuals. They are unique, and identification could be based on their comparison.[4]

However, the subjective nature of observation and interpretation within and between observers poses a problem. Lack of complete standardization in interpretation raises the validity of comparisons between different studies.

Despite the controversy about the stability of qualitative and quantitative characteristics of rugae and the extent of differences between ethnic groups and sex, the uniqueness to individuals has been recognized in forensic science as providing potential source of identification.[3]

The present study was undertaken to evaluate the qualitative and quantitative characteristics of rugae in 2 different populations. The total number of rugae in the MP and Kerala groups of population and between the 2 sides of the palate did not show any statistically significant difference. Dhoke and Usato (1994)[3,7,9] reported that the right side of the palate had fewer rugae than the left side. It was explained that this was due to the phenomenon of regressive evolution, dominating the right side of the palate. However, Shetty *et al*. (2005)[7] in their study on the population in Tibet and Mysore found no statistical difference in the total number of rugae between the races or the side of the palate.

Qualitative assessment of palatal rugae in the total subjects of both the groups showed that wavy pattern was predominant, followed by curved and then straight. This finding goes in accordance with Kapali *et al*. (1997);[3] in their study of Australian Aborigines and Caucasians, the most common shapes in both the ethnic groups were wavy and curved, whereas straight and circular were least common. On the contrary, in the present study we found that straight form was considerably high in number.

On comparing the 2 sides of the palate, right side showed a significantly more number of straight palatal rugae in males from MP, whereas wavy pattern was predominant in Keralite males. Furthermore, in the present study we found that unifications were moderate in number. Moreover, circular rugae were present in both the ethnic groups of population, although constituting only (4%) of the total rugae shapes. It was also noticed that circular pattern was more commonly seen as the first primary rugae anteroposteriorly [Figure 1]. Whereas in the study done by Preethi *et al*. (2007)[2] on Western and South Indian population, circular group was found to be absent and unifications were few in number. Thus the comparative studies showed varying patterns of palatal rugae shapes between the populations. As suggested this may be due to the apparent lack of systemic trends and the need for more comprehensive understanding by further studies.

## Conclusion

The uniqueness of rugae pattern in an individual is promising. In the present study, on comparing the 2 sides of the palate, rugae pattern on the right side of male subjects showed straight shape as significantly predominant in the MP group, whereas wavy shape was predominant in the Keralites. Similar patterns were seen in the total subjects of both the groups on the right side palate. Thus a statistically significant association between the rugae shape in the 2 populations exists although subtle. This requires further extensive study for establishing its significance in personal and racial identification.
